# Valorization of Soybean Meal: From Conventional Feed Ingredient to Multifunctional Protein Resource for Food, Biotechnology, and Sustainable Applications

**DOI:** 10.3390/molecules31183279

**Published:** 2026-09-16

**Authors:** Oanh Thi Kim Nguyen, Parushi Nargotra, Yung-Chuan Liu, Chia-Hung Kuo

**Affiliations:** 1 Faculty of Food Science and Technology, Ho Chi Minh City University of Industry and Trade, Ho Chi Minh City 700000, Vietnam; 2 Department of Seafood Science, National Kaohsiung University of Science and Technology, Kaohsiung 811, Taiwan; 3 Department of Chemical Engineering, National Chung Hsing University, Taichung 402, Taiwan; 4 Center for Aquatic Products Inspection Service, National Kaohsiung University of Science and Technology, Kaohsiung 811, Taiwan

**Keywords:** soybean meal, protein valorization, fermentation substrate, bioactive peptides, techno-functional properties, environmental sustainability

## Abstract

Soybean meal (SM) is a by-product of soybean oil extraction and represents one of the most abundant and economically important plant-derived protein resources worldwide. Traditionally utilized as a primary protein ingredient in animal feeding systems, SM has gained increasing attention in recent years due to rising global protein demand, food system pressures, and the need to reduce the environmental footprint of animal-based protein production. In addition to serving as a protein source, SM possesses intrinsic structural and biochemical features that underpin a wide range of functional and bioactive properties. These properties vary with processing conditions, particularly between high-temperature SM (HM) and low-temperature SM (LM). This review provides an integrative analysis of SM by connecting its compositional characteristics, molecular properties, bioactive peptides generation, structure–function relationships, and diverse food and industrial applications. Particular emphasis is placed on the production and biological activities of SM-derived bioactive peptides, processing-driven modulation of structural, functional, and bioactive properties, and emerging applications in human food formulations, industrial biotechnology, and bio-based materials. Agricultural and environmental applications are also discussed, including soil amendment, environmental management, and circular bioeconomy strategies. Finally, life-cycle assessment studies are reviewed to assess the environmental performance of SM-based systems and identify opportunities for improving resource efficiency and reducing greenhouse gas (GHG) emissions. By integrating perspectives from protein chemistry, bioprocessing, food science, industrial biotechnology and sustainability assessment, this work highlights the valorization of SM from a conventional feed ingredient into a multifunctional protein source for the development of high-value food ingredients and industrial bio-based products.

## 1. Introduction

The global human population is projected to reach approximately 10 billion by 2050, placing increasing pressure on food systems to provide adequate, reliable, and sustainable nutrition. This burden is further exacerbated by resource depletion, climate change, pandemics, and geopolitical disruptions that have exposed structural weaknesses in global food supply chains. Extreme climatic events such as floods, droughts, and wildfires increasingly threaten agricultural productivity, while socioeconomic inequalities and conflicts have contributed to volatility in global food prices and access. Amid these mounting challenges, the sustainability of protein production has become a central priority for ensuring long-term global food security [1]. Proteins are essential macromolecules that provide amino acids required for growth, tissue maintenance, and physiological processes. They also perform diverse biological functions, including structural support, enzymatic catalysis, transport, signaling, and immune defense [2].

The environmental footprint of protein supply is of particular concern due to the substantial contribution of livestock systems to GHG emissions. Livestock supply chains account for approximately 14.5% of global anthropogenic GHG emissions, corresponding to about 7.1 gigatons of CO_2_ equivalents. Meat and dairy production contribute nearly 65% of these emissions, a large proportion of which arises from methane release (45%) [3,4,5]. Modeling studies using the Integrated Science Assessment Model (ISAM) further indicate that GHG emissions from animal-based foods are roughly twice those from plant-based foods. Beef and rice serve as representative examples, contributing around 25% and 12% of emissions from animal- and plant-based foods, respectively [6]. At the same time, global consumption of meat, dairy, and cereals is projected to increase by 6.9%, 6.1% and 8.5%, respectively, driven by population growth and rising incomes. Given that animal products currently supply about 37% of global dietary protein, rising to nearly 58% in high-income regions, protein production plays a central role in ensuring both nutritional adequacy and environmental sustainability [7]. Alongside nutritional considerations, increasing attention has been paid to the valorization of agro-industrial co-products as multifunctional resources for the food and industrial sectors while supporting sustainability goals.

In response to these challenges, plant-derived protein sources have attracted increasing attention as more resource-efficient and environmentally favorable alternatives capable of supporting global protein demand while mitigating GHG emissions [8]. Among these, SM occupies a central position owing to its nutritional quality, large-scale availability, and broad applicability for agricultural, food, and industrial sectors. SM is the principal co-product of soybean oil extraction and is generated in substantial quantities as the solid fraction remaining after oil removal [9,10]. Global SM production has increased steadily with the expansion of the soybean oil industry, reaching hundreds of millions of tonnes annually and providing a stable, economically accessible protein supply [11,12]. Characterized by a high protein content, a balanced amino acid profile, and a favorable cost–performance ratio, SM has become one of the most widely utilized plant-based protein sources in animal nutrition, serving as a key component of poultry, livestock, and aquaculture feeding systems [13,14,15,16,17].

Although SM has traditionally been regarded primarily as a feed ingredient, its compositional complexity is not limited to crude protein provision. The major protein components of SM are 11S globulin (glycinin) and 7S globulin (β-conglycinin) [18]. Due to their inherent structural differences, these two globulins exhibit distinct physicochemical and functional properties [19]. In addition to essential amino acids, SM contains carbohydrates, dietary fiber, and diverse bioactive compounds, particularly isoflavones such as daidzein, genistein, and glycitein. These constituents contribute to functional properties, including improved nutrient utilization [20,21], modulation of gut health, and favorable physicochemical characteristics [22], thereby expanding the potential value of SM beyond conventional feed applications. Advances in processing technologies, such as fermentation, enzymatic hydrolysis, and conjugation approaches, have further enhanced the nutritional quality of SM by reducing antinutritional factors [23,24]. These technologies also generate bioactive peptides with improved physicochemical properties and biological activities, thereby strengthening the functional performance of SM [12,25,26]. The structural diversity of SM proteins provides a molecular basis for tailoring their functional properties and expanding their applications as value-added ingredients.

Several reviews have discussed SM in animal nutrition, fermentation, and bioactive peptide production. However, these reviews have generally focused on specific applications or processing approaches, with limited integration of the compositional, structural, functional, and industrial aspects of SM. This review addresses this limitation by providing an integrated perspective on the valorization of SM across food, biotechnology, agriculture, industry, and sustainability. Driven by sustainability imperatives and the need for improved resource efficiency, research interest in SM has progressively shifted from its conventional use as an animal feed ingredient toward broader valorization as a multifunctional protein resource. Despite increasing recognition of SM-derived proteins and peptides for food formulations, industrial biotechnology, and other emerging applications, the relationships among protein composition, structural characteristics, processing-driven modifications, and bioactive functionality remain insufficiently integrated. Therefore, this review provides an integrative analysis of SM by connecting its compositional and molecular characteristics with bioactive peptide generation, structure–function relationships, food and industrial valorization strategies, and life-cycle considerations.

### Literature Search Methodology

A structured literature search was conducted to identify studies addressing the composition, processing, biological functionality, valorization, applications, and sustainability of SM. Searches were performed using Google Scholar and PubMed, with soybean meal paired with terms related to its composition, processing, biological activities, and major applications, including composition, antinutritional factors, bioactive peptides, hydrolysis, fermentation, food, animal feed, aquaculture, poultry, livestock, adhesive, bioorganic fertilizer, soil amendment, composting, vermicomposting, environmental remediation, biosorption, life cycle assessment, sustainability, and valorization. The keywords were searched individually and in combination within the major areas of the review. No strict publication-year restriction was applied; however, emphasis was placed on recent peer-reviewed literature, particularly studies published between 2018 and 2026, while earlier studies were retained when they provided foundational evidence or addressed specific applications relevant to the review, particularly in biofertilizer development, enzyme production, and the characterization of bioactive compounds. Studies were selected based on their relevance, methodological quality, and substantive contribution to the topics discussed. The identified literature was then organized and discussed to provide an integrated perspective on SM, encompassing its conventional use in animal nutrition and its emerging applications in human food, biotechnology, agriculture, environmental management, and sustainability assessment. The literature search was updated in August 2026.

## 2. Classification and Main Compositions of SM

SM is the major co-product generated during soybean oil extraction. Its global production has increased in line with the growth of the soybean oil sector [27]. Global soybean production reached nearly 353 million tons, while soybean oil production reached 59.9 million tons in 2020 [28]. Based on the oil extraction process, SM is generally classified into high-temperature SM (HM) and low-temperature SM (LM) [29]. HM accounts for approximately 95% of total production [30,31] and is produced using intensive thermal desolventization, causing extensive protein denaturation and reduced protein solubility [27]. HM is brown in color, whereas LM, produced under relatively lower temperatures, is yellow-white due to limited thermal denaturation of proteins. The protein contents of LM and HM were 49.64% and 48.75% on a dry basis, respectively, while their fat, ash, and fiber contents were 0.71%, 6.60%, and 3.24% for LM and 0.75%, 6.80%, and 2.80% for HM, respectively [32]. Consequently, HM is difficult to utilize in food processing and is primarily applied in the feed industry, where its utilization value is comparatively lower [33,34]. In contrast, LM is obtained under milder desolventization conditions, which minimize protein denaturation and preserve solubility [27]. Therefore, LM is considered a higher-quality protein ingredient and is more suitable for value-added food applications, despite its lower production proportion [35].

Following oil removal, SM retains a high protein content, which contributes to its widespread use in feed, food and pharmaceutical-related applications, owing to its low cost, biodegradability, non-toxicity, and broad availability [36,37]. Accordingly, SM is widely recognized as the most common source of plant-derived protein due to its high protein content of approximately 45% on a dry basis [38], balanced amino acid composition, and high production yield [30]. In addition to proteins, SM contains notable levels of bioactive compounds, particularly isoflavones and soyasaponins. These compounds have attracted increasing attention due to their potential health benefits, as consumption of soy-derived bioactives has been associated with a reduced risk of chronic diseases, including cardiovascular diseases and certain types of cancer [39,40]. Carbohydrates represent another major compositional fraction of SM, accounting for approximately 30–35% on a wet weight basis. This fraction consists mainly of structural polysaccharides and galactose-containing oligosaccharides such as raffinose and stachyose [41]. A substantial proportion of these carbohydrates is present as dietary fiber (DF), which comprises about 15–20% of SM [42]. Although research attention has largely centered on SM proteins and derived peptides [43], DF remains a major yet comparatively underexplored component. Several recent studies have specifically investigated SM-derived dietary fiber (SMF). Dry fractionation of SM produced SMF with a looser and more porous structure, lower crystallinity, and significantly higher water-holding capacity, oil-holding capacity, and swelling ability than untreated SM. In addition, in vitro fecal fermentation of SMF promoted the production of short-chain fatty acids (SCFAs), including acetate, propionate, and butyrate, and increased the relative abundance of *Prevotella*, *Dialister*, and *Bifidobacterium*, while decreasing that of *Escherichia-Shigella* [22]. Moreover, insoluble DF from SM was converted into soluble DF through ball milling combined with enzymatic hydrolysis, achieving a conversion rate of 69.8% and generating soluble DF with distinct molecular and rheological properties [44].

In addition to its nutritional value, SM protein is increasingly recognized as a bioactive and multifunctional ingredient [45,46]. It exhibits favorable emulsifying and foaming properties, the ability to bind flavor compounds, and the capacity to act as a carrier for bioactive substances and nanomaterials [47]. Notably, SM protein isolates have been extensively investigated for their ability to form nano-emulsions and nanocomplexes, enabling the delivery of food-grade nanoparticles in advanced applications [48]. Despite these nutritional, functional, and economic benefits, the broader utilization of SM is constrained by antinutritional factors (ANFs) and its allergenic potential [49]. ANFs can hinder digestion, absorption, and utilization of nutrients, negatively affecting animal growth and human health [50,51,52]. Major ANFs in SM include trypsin inhibitors (TIs), phytate, antigenic proteins, lectins, saponins, tannins, non-starch polysaccharides (NSPs), and oligosaccharides (raffinose and stachyose). TIs, including Kunitz and Bowman–Birk and lectin, inhibit trypsin and chymotrypsin, thereby impairing protein hydrolysis and intestinal absorption, whereas phytate, saponins, tannins can interfere with mineral bioavailability and enzyme activity [13,53]. Furthermore, NSPs and oligosaccharides may increase digesta viscosity and cause osmotic distress in monogastric animals [53]. In addition, allergenic proteins (β-conglycinin and glycinin) are other ANFs affecting the use of soy protein products, which can induce symptoms ranging from skin, gastrointestinal, or respiratory reactions to anaphylaxis in human subjects [13]. To address these constraints, bioprocessing strategies such as fermentation, protein hydrolysis, and alternative-assisted technologies have been applied to improve the applicability of SM proteins.

**Figure 1:**
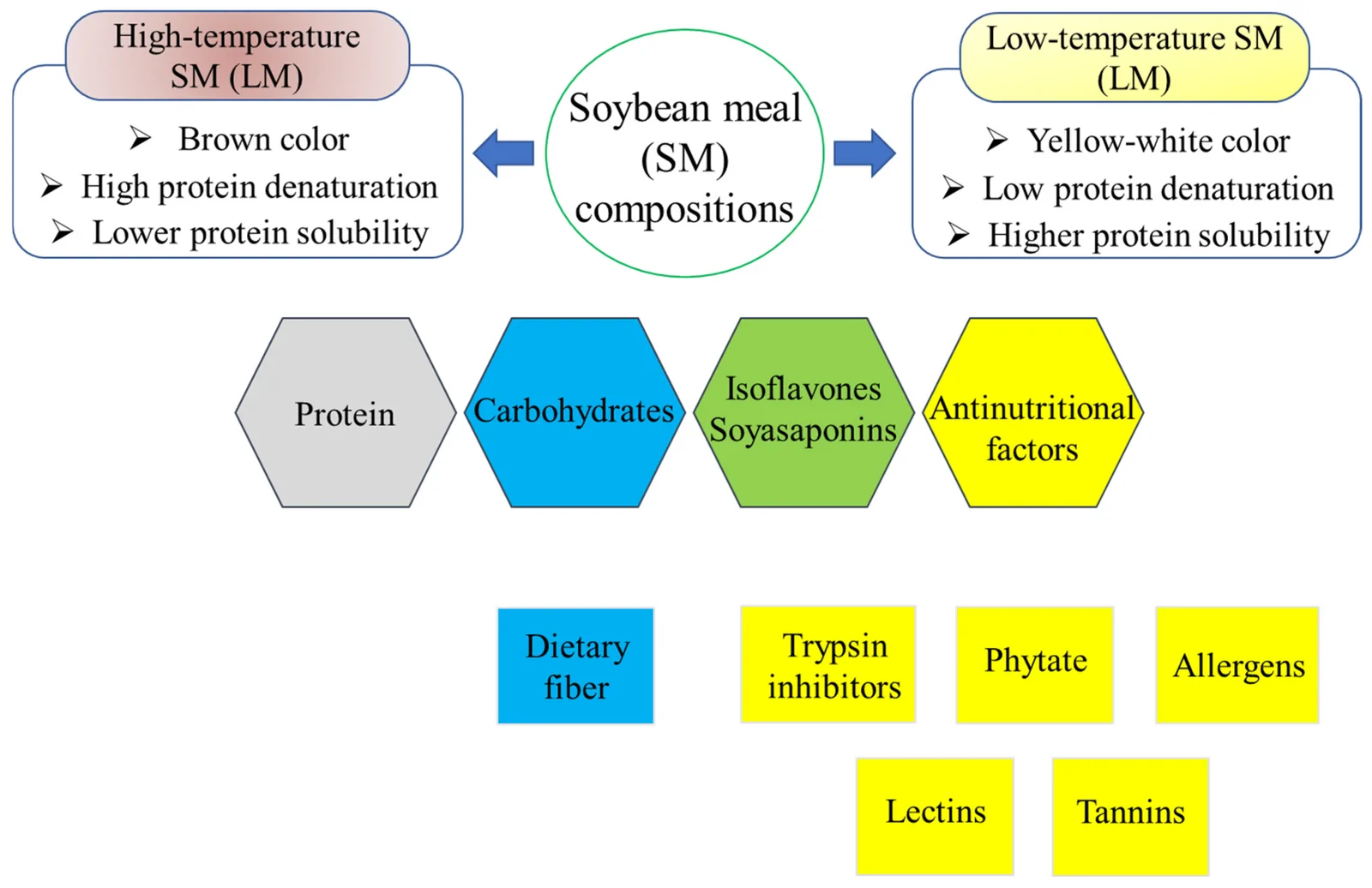
The main components of SM.

Taken together, these compositional characteristics position SM as a multifunctional ingredient that supports applications ranging from conventional animal feed to human food formulations, biotechnological processing, agricultural and environmental systems. These fundamental attributes provide a clear foundation for the subsequent sections, which examine breeding strategies, food development, industrial utilization, and life-cycle considerations within both traditional and advanced application contexts. The main components of SM are shown in Figure 1.

## 3. Breeding Applications of SM

**Figure 2:**
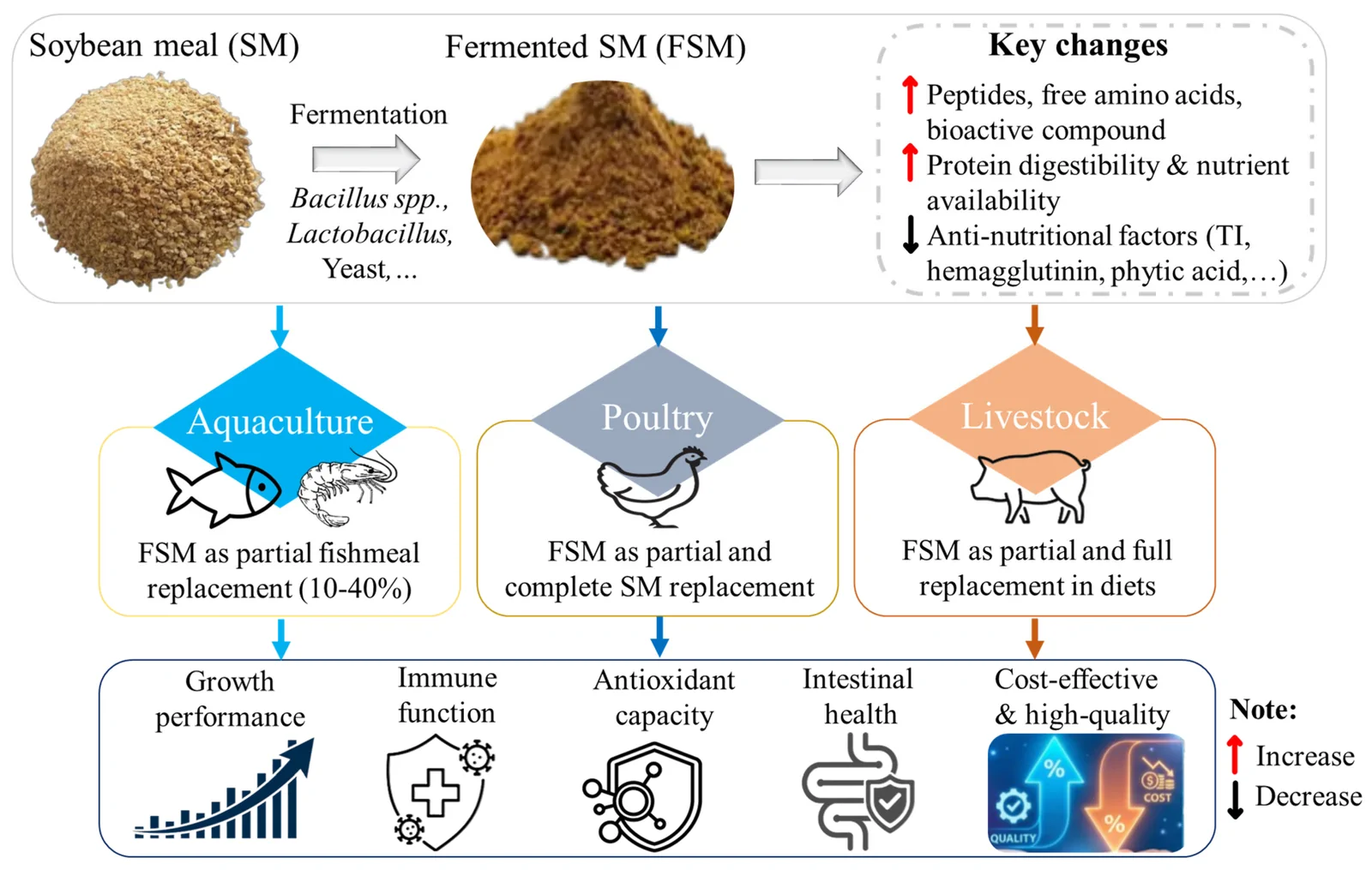
Schematic illustration of SM applications in breeding.

SM and its processed forms, particularly fermented variants, have been widely used in aquaculture, poultry, and livestock production for their nutritional value. Fermented SM has been reported to provide enhanced nutritional characteristics, including increased levels of peptides, free amino acids, and other bioactive compounds, while degrading antinutritional factors such as TI, hemagglutinin, polysaccharides, and phytic acid, thereby improving protein digestibility and overall nutrient availability [54]. The illustration of the breeding applications of SM is presented in Figure 2.

### 3.1. Aquaculture Field

Fishmeal (FM) has traditionally served as the primary protein source in aquaculture feeds due to its high nutritional value. However, the rapid growth of aquaculture, combined with overfishing and environmental pressures, has constrained FM supply and increased feed costs [55]. Consequently, the development of sustainable, cost-effective alternative protein sources has become a critical priority for the long-term development of the aquaculture industry. In this regard, SM has emerged as one of the most promising plant-based substitutes, owing to its wide availability, favorable amino acid profile, and economic competitiveness.

However, the nutritional and physiological effects of SM in aquaculture are strongly influenced by its processing condition, composition, and dietary inclusion level, as residual ANFs may adversely affect nutrient utilization and intestinal function [56].

Fermented SM (FSM) has been extensively evaluated as a partial FM replacement in aquafeeds. An eight-week trial in rainbow trout (*Oncorhynchus mykiss*), FSM was produced through a two-step solid-state fermentation (TS-SSF) process, initially with *Bacillus subtilis* (*B. subtilis*) and subsequently with *Lactobacillus* and *Saccharomyces cerevisiae* (*S. cerevisiae*). Up to 40% of FM could be replaced with FSM without impairing growth performance or feed utilization, although gut histological observations and serum biochemical indices suggested that a 20% replacement level may be more suitable [55]. In juvenile coho salmon, approximately 10% of FM protein replacement with *B. pumilus*-fermented SM improved growth, protein deposition, antioxidant and digestive enzyme activities, protein synthesis, and immune-related gene expression. The FSM was produced by solid-state fermentation (SSF), in which SBM was mixed with sterile water at a 1:1.2 (*w*/*w*) ratio, inoculated with 8% *B. pumilus*, and fermented for 36 h at 37 °C before drying. These findings highlight the potential functional benefits of FSM in aquafeeds [57]. Comparable benefits of FSM have also been reported in crustaceans and omnivorous fish species. In juvenile white shrimp (*Litopenaeus vannamei*), a 20% FM substitution with commercially produced FSM fermented using a mixed microbial culture of *S. cerevisiae*, *B. subtilis*, *Rhodopseudomonas palustris*, and *Bifidobacterium lactis* activated the TOR signaling pathway, with concurrent improvements in growth-related and digestive responses [58]. In Nile tilapia, Wang et al. reported that complete replacement of SM protein with FSM protein produced through SSF using *Pediococcus pentosaceus* YC64 (37 °C for 72 h at a SM-to-water ratio of 1:9 (*w*/*v*)) improved weight gain, feed efficiency, whole-body protein content, and amino acid utilization. FSM feeding also modulated muscle texture, promoted goblet cell proliferation, enhanced mucin secretion, strengthened intestinal tight junctions, reduced inflammatory responses, and increased *Pediococcus* abundance in the gut microbiota [59]. SM hydrolysates produced via SSF with *B. subtilis* Hs-2 37 °C/24 h enhanced nutritive value and beneficial metabolites [56]. Nevertheless, evidence also indicates that increasing FM substitution with processed SM may impair growth and intestinal morphology despite improvements in liver metabolism, as observed in rainbow trout with complete FM replacement with commercially available enzymatically hydrolysed SM [60], and in juvenile pearl gentian grouper when 20–40% of FM was replaced with commercial FSM [15]. Furthermore, in red seabream (*Pagrus major*), short-term feeding with defatted SM slightly suppressed trypsin secretion, whereas chronic feeding (six weeks) reduced bile storage, pancreatic enzyme production, and secretion, contributing to lower growth performance [61]. These findings indicate that fermentation can improve the nutritional and functional properties of SM by reducing certain ANFs and enhancing nutrient utilization and intestinal health. The variability in results among studies may partly be associated with differences in ANFs content and composition, SM type and processing history, together with dietary inclusion level, fish species, and experimental conditions. Therefore, optimizing the type, processing method, and inclusion level of SM or FSM remains essential for effective aquafeed formulation.

### 3.2. Poultry Breeding Field

Recent studies have explored the application of SM and FSM in poultry diets to enhance growth performance, nutrient utilization, and intestinal health.

SSF of defatted SM using *B. subtilis* ED-3-7 effectively enhanced the nutritional value of SM by degrading macromolecular antigenic proteins and ANFs. Feeding FSM to chickens improved growth performance, nutrient digestibility, and intestinal microbiota composition, with *Lactobacillus* emerging as the dominant genus [62]. Partial replacement of SM with 5% FSM produced through SSF significantly improved broiler growth, nutrient utilization, and feed efficiency, while inducing favorable changes in intestinal morphology, including decreased crypt depth and an increased villus-to-crypt ratio, indicative of enhanced absorptive capacity [63]. Abdel-Raheem et al. reported that double-fermented SM (DFSM), produced through double-SSF using *A. oryzae* and *B. subtilis* further enhanced broiler performance. Complete replacement of SM with DFSM improved body weight gain, feed conversion, and digestive enzyme activities. Moreover, DFSM feeding enhanced immune responses, antioxidant capacity, breast muscle nutritional quality, and increased intestinal lactic acid bacteria abundance [64]. Although comparative studies of one-stage and two-stage solid-state fermented SM remain limited, available evidence indicates that the additional fermentation stage does not necessarily produce major changes in several major proximate composition parameters but may modify specific nutritional and functional characteristics. In a comparative study, one-stage and two-stage solid-state fermented SM, produced with *B. velezensis* alone or in combination with *Lactobacillus*, respectively, showed no significant differences in several major compositional parameters, including crude protein, ether extract, ash, and total phosphorus. However, differences were observed in TCA-soluble protein, A-NSI, trypsin inhibitor content, and lactic acid bacteria abundance. Both fermentation strategies increased soy peptide content and free radical scavenging capacity and modulated distal gut microbiota and antioxidant-related gene expression during intestinal passage [16]. These findings indicate that the additional fermentation stage may influence specific nutritional and functional characteristics, particularly protein solubility, ANFs, and microbial properties, rather than substantially altering several major proximate composition parameters of SM. In Japanese quail, solid-state fermented SM exhibited probiotic-like effects comparable to those of commercial probiotic supplementation. Replacement of conventional SM with FSM reduced ANFs and increased nutritional quality. These changes were associated with enhanced growth performance, a more favorable balance of gastrointestinal microbiota in the crop and ceca, improved small intestinal morphology, and healthier serum lipid profiles [65]. These findings demonstrate that SM fermentation strategies, particularly multi-stage and strain-specific processes, can transform SM from a conventional protein source into a functional feed ingredient for poultry.

### 3.3. Livestock Breeding Field

SM is also widely used as a primary protein source in livestock feeds. Czech et al. reported that the dietary inclusion of FSM-based ingredients, either alone or in combination with fermented rapeseed meal, has been associated with improved intestinal morphology and microbiota composition, and nutrient digestibility, resulting in enhanced growth performance in weaned pigs [66]. SSF of SM produced with *B. subtilis* BS12 substantially reduced glycinin and β-conglycinin by 92.36% and 88.44%, respectively. Accordingly, piglets fed a diet containing 10% FSM demonstrated enhanced growth performance, which was attributed to reduced gut inflammation. These findings highlight the potential of FSM as a functional feed ingredient that can enhance gut health and reduce the need for antibiotics in pigs [67]. Another research indicated that FSM improved the nutritional value of diets for weaned pigs by increasing crude protein, amino acids, and lactic acid levels while reducing ANFs such as glycinin, β-conglycinin, and TI. These changes enhance protein and amino acid digestibility and support physiological functions, including intestinal integrity, digestive enzyme activity, antioxidant capacity, and immune response [17]. Deng et al. further demonstrated that FSM produced using *Bacillus* spp. could fully replace animal-derived protein supplements in diets for nursery pigs with an average body weight of approximately 11 kg, without compromising intestinal health or growth performance [68]. The functional benefits of FSM extend from the post-weaning stage to both maternal and finishing pigs. Luo et al. observed that replacing SM with solid-state fermented SM produced using *A. oryzae* and *Lactobacillus reuteri* in sow diets decreased oxidative stress during gestation and lactation, increased colostrum protein, fat, lactose, total solids, solids-not-fat, and immunoglobulin content, and enhanced the average daily gain of suckling piglets. These effects were partly linked to increased soybean isoflavones in the aglycone form [69]. In finishing pigs, dietary FSM produced through SSF using *B. subtilis* improved growth performance, nutrient digestibility, serum and muscle antioxidant capacity, and meat quality. A basal diet supplemented with 7.42% FSM resulted in changes in postmortem pH, redness, intramuscular fat content, and the expression of metabolism- and muscle fiber-related genes in the *longissimus thoracis* [70]. Similarly, a two-stage solid-state fermented feather meal-SM product (TSFP) included at 5% in finishing pig diets enhanced early feed intake, feed conversion, blood lipid profiles, growth performance, and immune-related indicators [71]. These observations demonstrate that fermentation of SM, either alone or in combination with other protein sources, enhances its nutritional value and functional properties across multiple stages of pig production. As a result, FSM supports improved growth performance, gut health, antioxidant status, immune function, and meat quality.

Collectively, these studies highlight SM and its fermented derivatives as versatile protein resources that support diversified breeding systems. Their successful application in aquaculture, poultry, and livestock production underscores the importance of processing strategies and formulation precision in maximizing nutritional efficiency. This approach also helps reduce reliance on conventional animal-derived proteins, which contributes to more sustainable breeding systems.

## 4. Human Food Applications of SM

Recent advances in processing and formulation strategies have enhanced the valorization of SM as value-added food ingredients by improving its nutritional value, sensory quality, and techno-functional performance. Processing approaches, including enzymatic hydrolysis, microbial fermentation, and protein modification strategies such as Maillard and polyphenol conjugation, induce molecular and conformational changes in soybean proteins, increasing the accessibility of functional groups and promoting beneficial intermolecular interactions. These structural modifications enhance solubility, interfacial properties, antioxidant potential, and other techno-functional attributes, thereby facilitating the development of diverse SM-derived functional food ingredients [21,72,73,74]. The applications of SM and SM-derived products in human foods are summarized in Table 1, while Table 2 provides an overview of the bioactivities and potential applications of SM-derived peptides and peptide fractions.

SM has been incorporated into food products as an ingredient. For example, biscuits formulated with extracted SM show increased protein content while maintaining sensory attributes such as sweetness, bean flavor, and crispness, and lowering the glycemic index [75].

Enzymatic hydrolysis represents a key approach for valorizing SM, as it modifies molecular structures and generates bioactive peptides. For example, alcalase is widely used to hydrolyze plant proteins, yielding peptides with specific biological activities. It is considered a “serine endopeptidase”, which cleaves proteins in the middle of the amino acid chain. Due to the wide range of amino acids it can recognize, alcalase-catalyzed protein hydrolysis tends to yield a hydrolysate with many small peptides [76]. Zhang et al. reported that alcalase-catalyzed hydrolysis of low-heated defatted SM produced low-molecular-weight peptides with antioxidant and ACE inhibitory activities [73]. Appropriate enzymatic hydrolysis also improves the sensory properties by reducing off-flavor compounds such as 1-octene-3-ol, thereby enhancing umami perception [77], while wheat malt-derived endopeptidase generates low-molecular-weight peptides (<3 kDa) with improved solubility and antioxidant capacity [78], underscoring the importance of enzyme selection and processing conditions in optimizing SM functionality.

Fermentation provides another approach for transforming SM into nutritionally enhanced and functionally diversified ingredients and food products. SM has been used as a substrate for the production of fermented foods. SM has been used as the sole substrate for soy sauce production. Co-fermentation with *A. niger* and *A. oryzae* yields gluten-free products with richer taste and enhanced umami [79]. In addition, steam-explosion pretreatment of defatted SM markedly enhances the characteristic umami taste and sauce-like aroma of the resulting soy sauce [80]. LSF of SM using *B. amyloliquefaciens* SWJS22, which co-produces β-glucosidase and proteases, increases total phenolic and flavonoid contents while generating < 3 kDa peptides, contributing to elevated antioxidant activity [21]. SSF using *B. licheniformis* YYC4 improves protein hydrolysis and nutritional quality without prior sterilization [81], whereas two-stage SSF (TS-SSF) overcomes pH-related microbial limitations, resulting in higher crude and soluble protein contents, reduced crude fiber and trypsin inhibitors, and improved antioxidant capacity and interfacial properties [82]. TS-SSF with *B. velezensis* followed by *Lactobacillus plantarum* (*L. plantarum*) further degrades glycinin and β-conglycinin, increases protein and mineral contents, and confers antibacterial activity [83]. Fungal and yeast fermentations similarly enhance digestibility and bioavailability; for example, *Neurospora crassa* SSF produces low-molecular-weight protein fragments (<14.4 kDa), increases free amino acids and in vitro digestibility [84], and *Saccharomyces pastorianus* (*S. pastorianus*), combined with protease hydrolysis, reduces anti-nutritional factors, generates antioxidant peptides, and releases minerals such as Fe^2+^ and Ca^2+^ with improved chelation, solubility, and bioavailability, supporting more efficient absorption in the human body [85]. SSF of SM with *L. paracasei* CRL 207 produces soy paste enriched in bioactive isoflavones and free amino acids. The resulting soy paste also serves as a probiotic carrier, supporting the development of plant-based functional foods for vegetarian and lactose-intolerant consumers [86]. Probiotic SSF with *B. subtilis* BS12 generates bioactive polysaccharide that enhances beneficial gut microbes, including *Bifidobacterium* and *Lactobacillus*, and support fructose and mannose metabolism [87]. Fermentation of SM with *S. cerevisiae* also converts isoflavone glycosides to aglycones, enhancing fiber and lipid content, antioxidant activity, and aglycone isoflavones [40]. Urinary metabolite analyses further indicate faster and more extensive isoflavone metabolism following consumption of biscuits [88].

Combined bioprocessing approaches have been shown to enhance the functional properties of SM. Fermentation of defatted SM with *B. subtilis* followed by short-term alcalase hydrolysis [89], green solvent-assisted hydrolysis using natural deep eutectic solvents (NADESs) or ionic liquids (ILs) with proteases [90], pretreatments such as ultrasound, microwave irradiation, high-pressure heating, and mechanical milling prior to enzymatic hydrolysis (Alcalase, Flavourzyme) [31] and ultrasound-assisted LSF of defatted SM with *B. subtilis* has been reported to improve the nutritional and functional properties of SM. In these LSF processes, SBM (20%, *w*/*v*) supplemented with KH_2_PO_4_ (1%, *w*/*v*), with or without corn gluten meal (2%, *w*/*v*), was sterilized at 121 °C for 20 min and inoculated with *B. subtilis* at 10%. These processes reduced antigenicity, increased peptide and amino acid contents, improved solubility, and enhanced antioxidant and ACE-inhibitory activities, while mitigating undesirable flavors [91,92].

Combined hydrolysis and fermentation approaches have also been applied to develop SM-based foods. yogurts prepared from SM treated papain hydrolysis in combination with *Lactiplantibacillus plantarum* [93] or *Bifidobacterium animalis* subsp. lactis [94] have been shown to improve the nutritional and sensory quality, including reduced beany flavor and bitterness, enhanced aroma- and flavor-related compounds, and increased amino acid content.

SM-derived peptides and protein fractions can also be further modified to improve nutrient delivery and functional properties. Lipophilic proteins (LP) from SM form complexes with vitamin B12, enhancing radical-scavenging activity while slowing gastric release and improving intestinal bioaccessibility, as evidenced during simulated digestion [95]. Similarly, peptides generated from SM hydrolysates produced through LSF with *A. oryzae* chelate Zn(II) to form stable SMP-Zn(II) complexes that withstand thermal processing and promote zinc release and absorption in vitro [96]. SM-derived components exhibit broader potential as carriers for bioactive micronutrients. In addition to peptide-based chelation, SMHs also interact with Zn through -NH, -COOH, and -OH groups, with hydrophobic and electrostatic forces contributing to complex stabilization. These SMHs-Zn complexes exhibit good solubility and support Caco-2 cell growth [97].

SM protein fractions can also be modified through Maillard and polyphenol conjugation to enhance both functionality and sensory properties. Maillard reaction products (MRPs) generated from xylose, cysteine, and SM peptide fractions improve antioxidant activity and confer desirable umami and meaty flavors, suggesting their use as flavor enhancers in functional foods [72]. Similarly, Maillard-type conjugates of SM protein isolate (SPI) prepared from low-temperature defatted SM with maltodextrin (MD) enhance emulsifying capacity and emulsion stability [98], while covalent conjugation of SPI with chlorogenic acid modifies protein structure to further improve emulsion performance and provide mechanistic insight into protein–polyphenol interactions [25].

**Table 1:** Applications of SM and SM-derived products in human food.

SM and Its Derivatives	Methods	Applications	References
SM hydrolysate (Defatted SM)	Enzymatic hydrolysis (Flavourzyme and Protamex) Maillard reaction	Flavor enhancer	[10]
SPI (Low-temperature defatted SM)	Isoelectric precipitation Covalent conjugation	Emulsion food systems	[25]
Fermented SM	Fermentation (*S. cerevisiae*)	Biscuits	[40]
SM hydrolysate (Defatted SM)	Ultrasonication Enzymatic hydrolysis (Alcalase) Non-covalent interaction	Emulsifier	[45]
SM hydrolysate (Defatted SM, low-heated)	Enzymatic hydrolysis (Alcalase) Conjugation	Emulsifier	[46]
SM peptides	Enzymatic hydrolysis (Alkaline protease and Flavourzyme) Maillard reaction	Flavour enhancer	[72]
SM hydrolysate (Defatted SM, low-denatured)	Ultrasonication Enzymatic hydrolysis (Alcalase) Covalent conjugation	Emulsifier	[74]
SM protein	High shear homogenization	Biscuits	[75]
SM	Fermentation (*Aspergillus oryzae*, *Aspergillus niger*)	Soy sauce	[79]
SM (Defatted SM)	Steam explosion	Soy sauce	[80]
Fermented SM	Fermentation (*S. cerevisiae*)	Biscuits	[88]
SM	Enzymatic hydrolysis (Papain and Acid protease) Fermentation (*Lactiplantibacillus plantarum*)	Yogurt	[93]
SM	Enzymatic hydrolysis (Papain)	Yogurt	[94]
SPI (Low-temperature defatted SM)	Isoelectric precipitation Maillard reaction	Food-grade emulsifiers	[98]
SM hydrolysate	Enzymatic hydrolysis (Alkaline protease and Flavourzyme) Maillard reaction	Flavor enhancer	[99]
SM peptides (Defatted SM)	Enzymatic hydrolysis (Alkaline protease and Flavourzyme) Maillard reaction	Meaty flavor enhancers	[100]
SM hydrolysate (Defatted SM)	Enzymatic hydrolysis (Alcalase) Conjugation	Emulsifier	[101]

**Table 2:** Bioactivities and potential applications of SM-derived peptides/fraction.

SM Sources	Peptide Sequence	Molecular Weight	Mechanism of Action	Bioactivity	Model	Potential Application	Obtained Results	Limitation	Ref.
SMH (Defatted SM, low-heated)	KFGW	<1 kDa	Activated the Keap1–Nrf2–HO-1 pathway	Antioxidant Cytoprotective	In vitro HEK-293 cell model	Functional foods, beverages, and healthcare fields	Free radical scavenging and protection against H_2_O_2_-induced oxidative stress via SOD enhancement, ROS/NO reduction, Bcl-2/Bax/Caspase-mediated apoptosis regulation, and Keap1-Nrf2-HO-1 activation	No in vivo or clinical validation	[12]
FSM Hydrolysate	-	<1 kDa	Modulated enzymatic and non-enzymatic antioxidant defense systems	Antioxidant	In vitro erythrocyte model	Functional foods	Inhibition of AAPH-induced erythrocyte hemolysis via ROS suppression, antioxidant enzyme regulation (SOD, CAT, GSH-Px), and MDA reduction	No in vivo or clinical validation	[24]
SMH (Defatted SM)	EEQEWPRKEEK	2434 Da	Direct free radical scavenging	Antioxidant	In vitro chemical antioxidant assays	Natural antioxidant in food and medicine industries	The radical scavenging activity on DPPH free radical of fraction C3b was 33.57%	No in vivo or clinical validation	[102]
SM Proteins (Defatted SM)	-	Mw < 5 kDa (1.7–3.1 kDa)	-	Antibacterial	In vitro chemical antibacterial assays	Food and biochemical process industries	Against *Bacillus cereus*, with inhibition zones of 15 and 10 mm	No in vivo or clinical validation	[103]
FSM hydrolysate	QC GPNAV PNAV	-	ACE inhibition and endothelium-independent relaxation	Vasorelaxation	Ex vivo aortic ring	Food and/or drug	EC50 of vasorelaxation: 0.41–1.8 μM ACE inhibition ratio: 13.05–80.66%	No in vivo or clinical validation	[104]
FSM	PFGAGRRICAGLSLGLQMVQLLT HFDSEVVFF LFGDKPVTIF MLHIPVSVSTPGKF VVDMNEGALFLPH	-	Direct free radical scavenging	Antioxidant	In vitro chemical antioxidant assays	A functional food additive and/or nutraceuticals	% of antioxidant amino acids: 13.04–44.44	No in vivo or clinical validation	[105]
SMH	GTYW	-	Modulation of glutathione and metabolic pathways	Hepatoprotective (anti-ALD)	In vivo mouse model	Liver disease and functional food production	ALD prevention by preserving liver histology and regulating GSH, amino acid, and alcohol metabolism pathways	No clinical validation	[106]

## 5. Biotechnological and Industrial Applications

**Figure 3:**
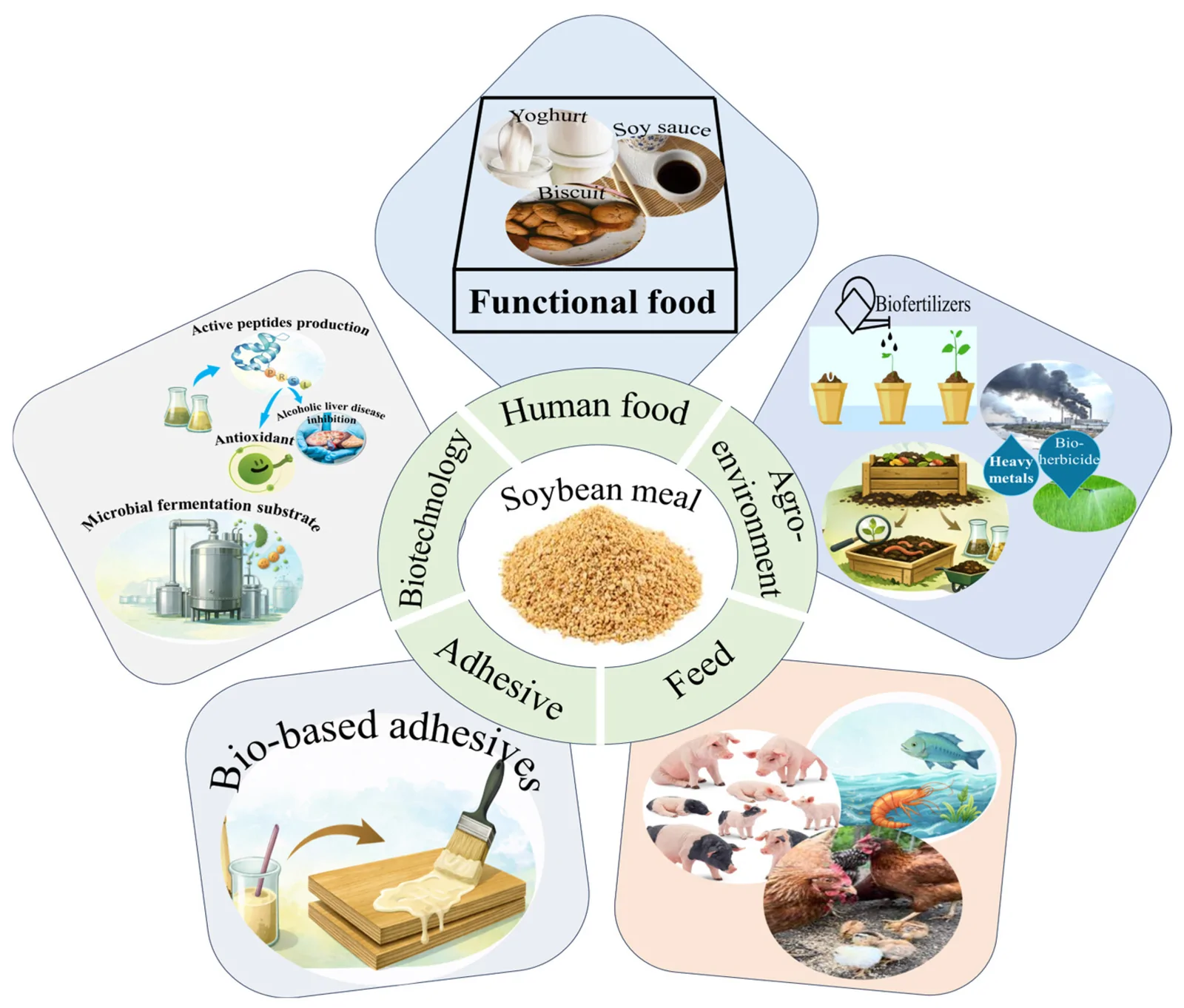
The schematic illustration of SM applications.

In addition to its traditional role as a protein-rich feed ingredient, SM has gained recognition as a multifunctional and sustainable raw material for a broad range of biotechnological and industrial applications. Owing to its high protein content, balanced amino acid profile, residual carbohydrates, and functional side components, SM serves as an effective substrate for biological transformation and value-added utilization [107]. Advances in biotechnology have enabled the conversion of SM into high-value products through enzymatic, microbial, and physicochemical approaches, thereby expanding its utilization. Building on these technological advances, SM has been increasingly explored for the production of bioactive peptides, as a low-cost substrate for microbial fermentation, and as a renewable feedstock for bio-based adhesive systems [108,109,110]. Collectively, these applications underscore the role of SM in linking conventional feedstock utilization with advanced industrial biotechnology. The schematic illustration of SM applications is depicted in Figure 3.

### 5.1. Bioactive Peptide Production

Bioactive peptides are short protein fragments composed of 2–20 amino acid residues with molecular weights below 3 kDa [111]. The discovery and characterization of bioactive peptides is therefore regarded as a promising strategy for the high-value utilization of protein sources [12]. In this context, SM represents a functional protein reservoir for the preparation of bioactive peptides intended for human consumption [91]. SM-derived peptides are predominantly low-molecular-weight fractions generated through proteolytic cleavage of storage proteins. These peptides generally exhibit enhanced biological activity, highlighting SM’s suitability as a substrate for producing peptide-based functional ingredients. Natural fermentation represents an effective strategy for converting SM proteins into low-molecular-weight bioactive peptides through the coordinated action of indigenous microbial consortia. During SSF, microbial proteases progressively hydrolyze soluble proteins, thereby resulting in the degradation of high-molecular-weight storage proteins, including β-conglycinin, Gly m Bd 30 K, and the acidic and basic subunits of glycinin, as confirmed by the gradual disappearance of their SDS-PAGE bands. In this process, SBM was moistened with sterile distilled water and incubated at 80% relative humidity at 30–55 °C, with moisture levels of 40–64% evaluated for optimization. Consequently, peptides with molecular weights below 3 kDa accumulate substantially, with the 1000–3000 Da fraction becoming predominant during fermentation, accompanied by increased peptide and free amino acid contents. Among the indigenous microorganisms, *B*. *subtilis* plays a central role by exhibiting strong positive correlations with protease activity and peptide production while simultaneously reducing trypsin inhibitor activity. These findings indicate that microbial fermentation not only remodels the protein structure of SM but also enhances its nutritional quality by generating peptide-rich fractions with potential bioactive properties [112]. In addition to indigenous microbial consortia, *Bacillus* strains isolated from traditionally fermented foods, such as kinema, have also been reported to efficiently convert SM proteins into functional peptides, further demonstrating the effectiveness of microbial fermentation for peptide production. During fermentation, radical-scavenging activity gradually increased with fermentation time, suggesting the progressive release of antioxidant peptides through microbial proteolysis. Peptidomic analysis identified a total of 1837 peptides from fermented SM produced using different starter strains, with *B. subtilis* KN36D generating the highest number of peptides, followed by *B. licheniformis* KN1G and *B. amyloliquefaciens* KN2G. Five novel antioxidant peptides (PFGAGRRICAGLSLGLQMVQLLT, HFDSEVVFF, LFGDKPVTIF, MLHIPVSVSTPGKF, and VVDMNEGALFLPH) were further identified, highlighting the capacity of *Bacillus*-mediated fermentation to generate bioactive peptides with antioxidant potential. The antioxidant activity of these peptides has been attributed to the presence of hydrophobic and aromatic amino acid residues, including proline, leucine, histidine, isoleucine, methionine, phenylalanine, tryptophan, and tyrosine [105]. In parallel with microbial fermentation, enzymatic hydrolysis also generates defatted SM-derived peptides with molecular characteristics associated with diverse biological activities. Hydrolysates enriched in short-chain peptides, particularly di- and tripeptides, exhibit diverse biological activities, including angiotensin-converting enzyme (ACE) inhibition, dipeptidyl peptidase IV (DPP-IV) inhibition, antioxidant activity, renin inhibition, and stimulatory effects. The antioxidant activity of these peptides has been linked to the presence of specific amino acid residues, particularly tyrosine-containing sequences such as (I/L)Y, VY, and AY, which contribute to enhanced free-radical scavenging capacity [113]. The influence of molecular size on the bioactivity of SM-derived peptides has been demonstrated through peptide fractionation studies. SM peptides separated into different molecular-weight ranges (<1, 1–3, 3–5, and >5 kDa) exhibited distinct antioxidant capacities, with the <1 kDa fraction showing the strongest activity. This fraction displayed antioxidant activity in chemical assays, as evidenced by DPPH (SC_50_ = 0.2517 mg/mL) and ABTS (93.83% scavenging at 0.5 mg/mL; SC_50_ = 0.2433 mg/mL), comparable to the positive control. The superior antioxidant capacity of low-molecular-weight peptides has been attributed to the greater exposure of antioxidant-active groups. Furthermore, SM-derived peptides effectively alleviated AAPH-induced oxidative stress in erythrocytes by inhibiting ROS generation and regulating intracellular antioxidant enzyme activities. These peptides also formed a protective layer around erythrocyte membranes, thereby reducing AAPH-induced cellular damage [26]. In addition to general antioxidant effects, specific peptides derived from defatted SM with defined sequences have been identified and mechanistically characterized. The tetrapeptide Lys-Phe-Gly-Trp (KFGW, <1 kDa), isolated from SM hydrolysates, exhibits strong radical-scavenging capacity and pronounced cytoprotective effects in HEK-293 cells exposed to oxidative stress. Its bioactivity is linked to increased SOD activity, reduced intracellular ROS and nitric oxide levels, and modulation of apoptosis-related signaling through the Bcl-2/Bax/Caspase pathway. Molecular modeling further revealed that KFGW can activate the Keap1-Nrf2-HO-1 pathway through hydrogen bonding and van der Waals interactions. These findings highlight the potential application of SM-derived peptides in antioxidant-enriched foods and health-promoting products [12]. SM is also a source of bioactive peptides that have been associated with potential effects on liver health and alcohol-related liver disease (ALD). Among the identified peptides, GTYW (Gly–Thr–Tyr–Trp) exhibited the strongest antioxidant activity, with an IC_50_ of 0.23 ± 0.09 μM, indicating potent free-radical scavenging capacity at a very low concentration. Integrated network pharmacology, proteomic, and in vivo analyses further demonstrated that GTYW has been reported to modulate signaling pathways associated with ALD, suggesting its potential application as a functional ingredient for supporting liver health. The remarkable antioxidant activity of GTYW is closely associated with its structural characteristics and amino acid composition. The peptide possesses a negative GRAVY value (−0.83), indicating high hydrophilicity, which may facilitate its dissolution in aqueous biological environments and improve its transport to target tissues. In addition, the presence of glycine, threonine, tyrosine, and tryptophan is likely to contribute synergistically to its antioxidant properties. Glycine and threonine have been reported to participate in antioxidant peptide activity, whereas the aromatic residues tyrosine and tryptophan are well recognized for their hydrogen-donating and radical-scavenging abilities. Furthermore, threonine and tryptophan are essential amino acids involved in maintaining physiological functions, and peptides containing these residues have been suggested to protect hepatocytes against oxidative stress and promote cellular repair [106].

### 5.2. Microbial Fermentation Substrate

In addition to its role as a precursor for bioactive peptide production, SM has been widely exploited as a fermentation substrate in diverse biotechnological processes. Its high protein content, balanced amino acid profile, and residual fermentable carbohydrates provide a favorable nutritional matrix that supports microbial growth, metabolic activity, and product biosynthesis. These intrinsic characteristics enable SM and its derivatives to function as low-cost, sustainable substrates for value-added fermentation systems.

In SSF, SM has demonstrated strong suitability for extracellular enzyme production. For instance, *Penicillium* P58 exhibited high lipase activity when cultivated on SM supplemented with urea and soybean oil, illustrating the capacity of SM-based substrates to support enzyme biosynthesis under nutritionally optimized conditions [114]. The functionality of SM as a fermentation substrate is further expanded through hydrolysis. SMHs have been successfully applied as an alternative nitrogen source in seed culture media, where they exerted a regulatory effect on fungal morphology. In *Rhizopus oryzae* ATCC 20344, SMHs promoted the formation of uniformly dispersed mycelial clumps (~0.1 mm), which improved mass transfer efficiency during fermentation and significantly enhanced fumaric acid production. This example illustrates how SMHs can simultaneously improve fermentation performance and valorize SM by-products [115]. Beyond organic acid production, SMHs have also been explored as substrates for bioethanol fermentation. Although ethanol is traditionally derived from sugar cane, corn, or beets, fermentable sugars released from SM hydrolysates can be efficiently converted to ethanol by *S. cerevisiae* and *Zymomonas mobilis*, which provides the ecological and economic advantages of utilizing SM-derived carbohydrates as an alternative feedstock [116]. SMHs further support microbial polysaccharide biosynthesis. In pullulan production, SMHs serve as a nutrient-rich source for *Aureobasidium pullulans* NCPS2016, thereby enhancing pullulan synthesis and reducing reliance on expensive conventional media. This strategy addresses the traditionally high production cost of pullulan and aligns with its desirable properties, including film-forming ability, water solubility, and biodegradability [117]. Similarly, SMHs have been shown to support efficient chitosanase production by *B. subtilis*. The use of SM-derived hydrolysates improved enzyme activity and yield while reducing overall production costs [118]. In addition to microbial and enzymatic bioprocesses, SM have been evaluated as substrates for edible and medicinal mushroom cultivation. When applied to the cultivation of *Hericium erinaceus*, SM-based substrates provided essential nutrients, including high-quality protein, dietary fiber, vitamins, and minerals, thereby promoting robust fungal growth. Notably, cultivation on SM-based substrates enhanced the accumulation of triterpenoids and total phenolics and increased antioxidant activity. This observation demonstrated that SM could modulate both biomass production and the bioactive profile of cultivated mushrooms [119]. Tetramethylpyrazine (TTMP) is a therapeutically relevant bioactive compound, and microbial fermentation using *Bacillus* sp. TTMP20 represents an efficient production approach. The use of SM as an alternative nitrogen source, in combination with molasses as the carbon source, significantly enhances TTMP yield and underscores the value of low-cost agro-industrial by-products for scalable TTMP biosynthesis [120]. SM and SMHs serve as fermentation substrates that support diverse bioprocesses, ranging from enzyme and organic acid production to the production of bioethanol, polysaccharides, functional mushrooms, and bioactive metabolites. Their application not only enhances fermentation efficiency and product yield but also reduces costs and enables the sustainable utilization of a widely available agricultural by-product in biotechnological systems.

### 5.3. SM-Based Adhesives

SM has also been explored as a renewable raw material for bio-based adhesive systems, driven by its high protein content and the presence of reactive functional groups. These characteristics enable the development of sustainable adhesive formulations while reducing dependence on petroleum-derived materials. To overcome the inherent limitations of SM-based adhesives (SMA), particularly insufficient water resistance and brittleness associated with low crosslinking density, multiple modification strategies have been proposed. Li et al. reported that ultrasound-assisted chemical modification of SMA using zinc oxide (ZnO) and polyamide epichlorohydrin resin (PAE) significantly enhanced adhesive performance. The improvement was attributed to ultrasound-induced protein cross-linking and strengthened interfacial interactions with wood substrates, resulting in a wet shear strength of 1.21 MPa—72.86% above the China National Standard and 19% higher than chemical modification alone. These findings establish ultrasonic-chemical treatment as a clean and efficient strategy for improving the performance of SM-based plywood adhesives [121]. In addition to ultrasonic modification, functional polyurethane-based crosslinkers provide another avenue for enhancing SM adhesives. Cationic waterborne polyurethanes with hydrophobic alkyl chains and catechol groups have been synthesized via thiol-ene click chemistry and direct emulsification. In these systems, catechol moieties facilitate hydrogen bonding with protein molecules to reinforce the cross-linked network, while hydrophobic chains and microphase-separated domains restrict moisture penetration and enable energy dissipation. This combination significantly improves wet bonding strength and toughness, representing a cost-effective and environmentally friendly strategy for producing high-performance SM-based adhesives [122]. Further improvements have been achieved through dual crosslinking strategies that restructure protein–polysaccharide networks. Calcium lignosulfonate (CLS) unfolds protein structures and forms reversible networks with protein and polysaccharide chains, which synergize with 1,6-hexanediol diglycidyl ether (HDE) to establish a compact, robust dual network. This configuration enhances bonding strength, increases residual rate (82.4%), reduces moisture uptake (6.85%), and improves mildew resistance and flame retardancy [123].

SM-based adhesives offer a sustainable alternative to petroleum-derived systems but are constrained by limited water resistance and brittleness arising from low crosslinking density. The incorporation of a flexible, hyperbranched long-chain starch with a suitable crosslinking agent increases crosslinking density and improves toughness, thereby enhancing both water resistance and mechanical performance. Plywood bonded with the modified SMA exhibits higher wet bond strength and meets interior-use requirements in pilot-scale production, confirming its practical applicability [124]. Adhesive performance of SM in wood composites is governed by the different types of SM, which control the availability of reactive groups and the resulting crosslinking density with epichlorohydrin-modified polyamidoamine (EMPA). Comparative evaluation of low-temperature (LM), high-temperature (HM), and physical (PM) SMs demonstrate that LM-based adhesives exhibit the highest bond strength and water resistance, satisfying the requirements for both interior-use and floor-base plywood. In contrast, adhesives derived from HM show slightly lower water resistance, although their performance approaches the standard threshold (>0.8 MPa) for floor-base plywood. These results indicate that LM is directly applicable for high-performance plywood production, while targeted modifications to enhance the reactivity of HM could extend its suitability to more demanding structural applications [27].

Through complementary approaches involving physical modification, chemical crosslinking, polymer integration, and material selection, SMA can achieve mechanical strength, water resistance, and durability comparable to conventional systems, supporting their transition toward industrial-scale wood bindery applications.

## 6. Agricultural and Environmental Applications of SM

**Figure 4:**
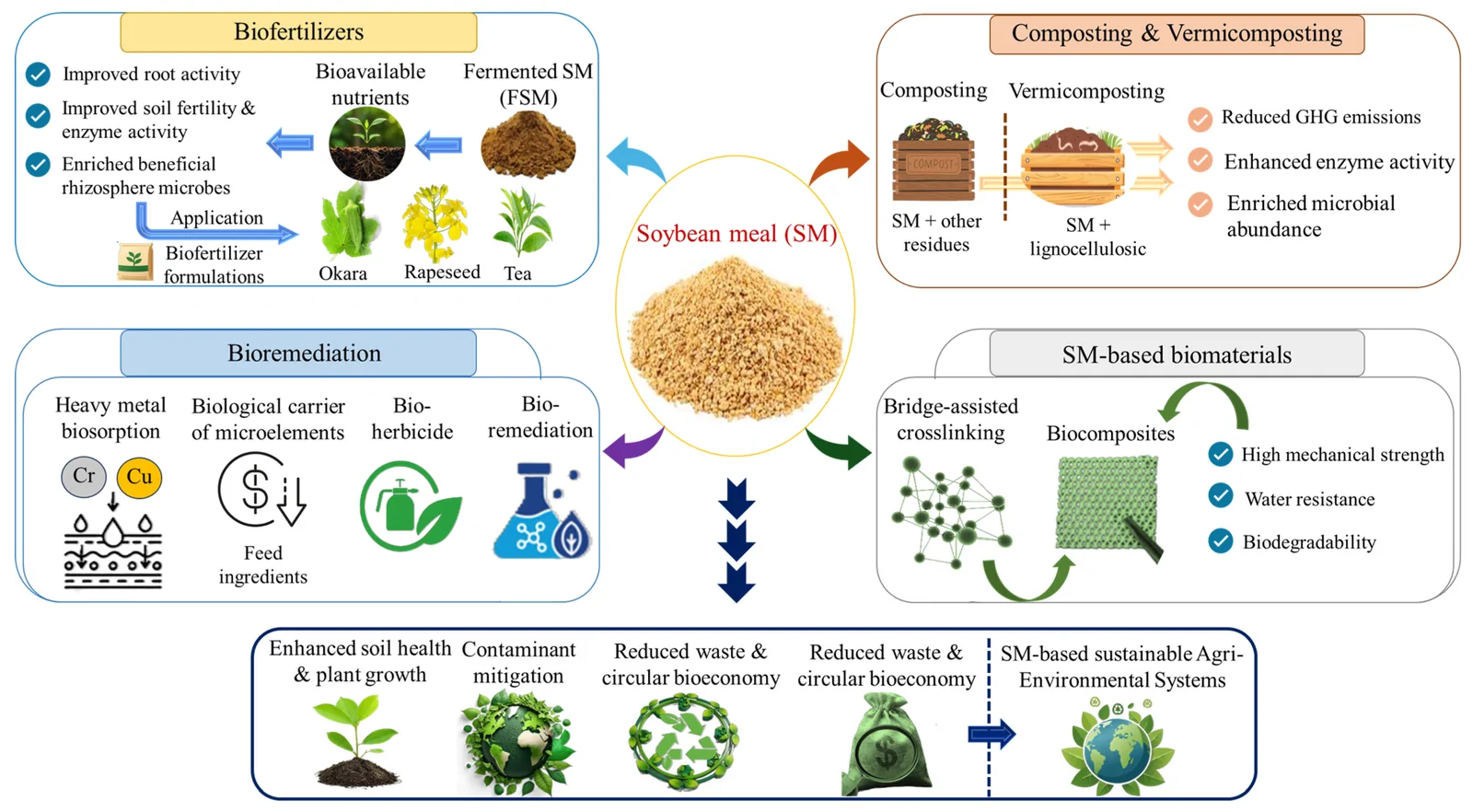
The schematic illustration of agricultural and environmental applications of SM.

SM, traditionally regarded as a low-value agro-industrial by-product, has increasingly been recognized as a multifunctional resource for agricultural and environmental applications. Biological, physicochemical, and materials-based transformations enable the valorization of SM into functional fertilizers, soil amendments, biosorbents, bioremediation agents, and bio-based materials. Recent studies demonstrate that tailored modification strategies facilitate the contribution of SM and its derivatives to sustainable crop production, soil health improvement, contaminant mitigation, and environmental management. Figure 4 provides a schematic representation of agricultural and environmental applications of SM.

### 6.1. SM-Based Biofertilizers and Soil Amendments

Biological transformation via fermentation has emerged as an effective strategy to convert SM into bioavailable nutrient sources for crop production. Compared with fermentation of SM for animal feed or human consumption, which is generally intended to improve nutritional quality, reduce ANFs, and/or enhance sensory or techno-functional properties, fermentation for bioorganic fertilizer and soil amendment focuses more specifically on the transformation of organic components to improve nutrient availability and agronomic value. SSF using the high protease-producing *B. subtilis* strain N-2 converts commercially available SM into a water-soluble fertilizer enriched in low-molecular-weight peptides and free amino acids. Application of this fermented fertilizer at a low dosage (0.25%, *w*/*v*) significantly enhanced rapeseed growth, as evidenced by increased root system activity, chlorophyll content, leaf area, shoot dry weight, and root biomass [125]. Another study indicated that FSM also exerts broader soil-conditioning effects, particularly in perennial cropping systems. In tea plantations, FSM application increased essential nutrients, including total phosphorus and trace elements (Cu, Zn, and Mn), together with amino acids and short-chain fatty acids. Concurrently, FSM enriched beneficial rhizosphere microorganisms such as *Pseudomonas* and *Bacillus*, stimulated soil enzyme activities, and improved soil fertility. These changes resulted in enhanced accumulation of amino acids, terpenoids, and other flavor-related metabolites in tea shoots, supporting both soil health and product quality [126]. Agronomic benefits of SM-based amendments have also been observed under conditions of reduced reliance on synthetic fertilizers. In okra cultivation, the use of SM resulted in improved plant growth and yield, characterized by increased fruit production, plant height, and a reduced time to 50% flowering, especially when conventional NPK fertilization was limited [127]. In parallel, formulation strategies combining SM with complementary substrates have enabled the development of scalable biofertilizers. The combined use of defatted SM and molasses supported the enrichment of biofertilizer formulations with *Enterobacter hormaechei* 40a, which provides a practical strategy for large-scale production and agronomic deployment of SM-based biofertilizers [128].

### 6.2. Composting and Vermicomposting for Soil Quality Enhancement

The valorization of food processing residues through composting and vermicomposting has been extensively explored to generate nutrient-rich products suitable for agricultural and horticultural applications [129]. Within this context, SM has been incorporated into composting and vermicomposting systems as a functional organic component to improve soil amendments. Composting represents an effective approach for agricultural waste management, with benefits for crop productivity and soil quality. A composted composite derived from SM, corn starch residue (CSR), and oyster shell powder (OSP) enabled controlled nutrient release, thereby supporting plant growth, as demonstrated in Chinese cabbage [130].

Vermicomposting has been reported to outperform conventional composting by reducing GHG emissions and enhancing the nutrient content of the end product [131]. Cai et al. further showed that combining SM with sugarcane bagasse markedly improved vermicompost quality by promoting mineralization and nitrification, increasing microbial abundance and enzyme activities, and enriching humic substances, while simultaneously supporting the growth and reproduction of earthworm (*Eisenia fetida*) [132]. Together, these results underscore the synergistic role of SM in stabilizing organic residues and enhancing the agronomic value of compost- and vermicompost-based soil amendments.

### 6.3. Environmental Remediation and Biosorption Applications

In environmental applications, SM has been widely investigated as a low-cost biosorbent for heavy metal removal. SM waste effectively removes Cr(III) and Cu(II) through ion exchange, coordination with carboxyl and hydroxyl groups, and surface precipitation [133]. Detailed analyses of chromium biosorption further confirmed the involvement of carboxyl, hydroxyl, and amino functional groups in Cr(III) binding, supporting the mechanistic basis for SM-based biosorption processes [134]. In addition to contaminant removal, biosorption has also been exploited to enrich SM with essential trace elements. Using a fixed-bed column system, Anna Witek-Krowiak et al. demonstrated the enrichment of SM with Cu(II) and Cr(III), transforming SM into a biological carrier of micronutrients. Owing to its low cost and strong sorption capacity, the resulting microelement-enriched SM showed potential as a feed ingredient, particularly for growing hens, by providing supplemental essential minerals [135]. From a broader environmental perspective, the use of SM as a dietary protein source may also have implications for the elemental composition of livestock manure. Although FM replacement with SM has been widely studied in pigs and poultry, direct evidence that such replacement reduces heavy-metal excretion in manure remains limited. As dietary trace elements can be transferred to manure and subsequently to agricultural soils, controlled studies comparing FM- and SM-based diets are needed to assess heavy-metal accumulation in manure and the subsequent transfer of these elements to soils and crops. SM derivatives have also been explored for environmentally benign weed management. SMH was prepared by suspending the sieved SM powder in deionized water to obtain a 10% (*w*/*v*) solution, followed by alkaline protease hydrolysis at 60 °C and pH 8.0 for 6 h. SMH demonstrated natural pre-emergence herbicidal activity by inhibiting radicle growth in germinating *Lolium perenne* seeds under non-sterile conditions. This effect was attributed primarily to free ammonia release rather than specific peptide activity, which highlights SMH as an environmentally friendly bioherbicide derived from soybean by-products [136]. Additionally, waste-derived amendments have been evaluated for the bioremediation of oil-contaminated soils due to their nutrient richness and cost-effectiveness. Recent studies in desert soils from Oman showed that SM enhanced the activity of hydrocarbon-degrading bacteria and promoted petroleum contaminant degradation. Although its stimulatory effect was lower than that of substrates such as sewage sludge or wheat straw, SM nonetheless demonstrated suitability as a cost-effective nutrient source for bioremediation applications [137].

In addition to its roles in fertilization, soil improvement, and environmental remediation, SM has been valorized as a bio-based material through targeted physicochemical modification. Kan et al. indicated that incorporating a DETA-bridge-modified MUG (DETA@MUG) crosslinker enhanced the structural integrity and performance of SM-based biocomposites. When combined with PAE resin, DETA@MUG promoted efficient co-crosslinking via protein–protein and protein–carbohydrate interactions, resulting in dense, interconnected network structures. These reinforced networks improved mechanical strength, water resistance, and biodegradability, thereby supporting the development of SM-based polymers and biocomposites with balanced functional properties via bridge-assisted crosslinking approaches [138].

These studies demonstrate the versatility of SM and its fermented, hydrolyzed, and modified derivatives in environmental applications, including heavy-metal biosorption, trace-element enrichment, weed management, soil bioremediation, and the development of bio-based materials. Despite this broad potential, several aspects of the environmental fate and safety of SM-derived applications remain insufficiently explored. In particular, the effect of dietary SM replacement on heavy-metal excretion in livestock manure and the subsequent transfer of these elements to agricultural soils and crops warrants further investigation within circular bioeconomy systems.

Collectively, these processing approaches induce distinct changes in the molecular and functional characteristics of SM. Fermentation can promote proteolysis, increase small peptides, and reduce certain ANFs, whereas enzymatic hydrolysis generates lower-molecular-weight peptides and free amino acids. In contrast, physicochemical crosslinking primarily modifies intermolecular interactions and network structure. These differences may contribute to the distinct functional properties and applications of SM derivatives.

## 7. Life-Cycle Assessment of SM

Life-cycle assessment (LCA) is an analytical tool used to comprehensively quantify and interpret the environmental exchanges between a product system and the environment throughout its entire life cycle (or process or service), including emissions to air, water, and land, as well as the consumption of energy and other material resources over the entire life cycle of a product (or process or service). By accounting for impacts across the product life cycle, LCA provides a comprehensive perspective on environmental burdens and trade-offs, enabling more informed comparisons among feed ingredients, production strategies, and formulation choices [139].

Recent life-cycle-based feed formulation analyses demonstrate that variations in SM composition can significantly influence both environmental and economic performance in poultry and swine diets. Increasing SM crude protein (CP) concentration from 44.0% to 48.0% was associated with higher SM valuation, corresponding to increases of approximately USD 19 and USD 16 per metric ton of feed in poultry and swine diets, respectively. Importantly, these compositional improvements simultaneously reduced diet-related GHG emissions by about 5.5% in poultry and 4.8% in swine production systems. These emission reductions coincided with changes in diet formulation, including increased use of corn grain and reduced SM inclusion rates, indicating that improved protein quality can enhance formulation efficiency. From a life-cycle perspective, higher amino acid and energy concentrations in SM improved the environmental performance of poultry and swine diets without increasing nitrogen excretion, which remained unchanged across different SM CP levels. Economically, higher-CP SM reduced total diet costs while lowering GHG emission intensity per kilogram of production, although formulations optimized strictly for minimum GHG emissions, particularly in swine diets, may increase feed costs by an estimated USD 6.50 per metric ton. These findings highlight the importance of incorporating ingredient quality, formulation strategies, and economic constraints within LCA-based evaluations of SM sustainability [140]. Dalgaard et al. conducted a consequential LCA to assess the environmental impacts of SM consumption, defining the functional unit as one kilogram of SM produced in Argentina and delivered to Rotterdam Harbor. The consequential approach applied system expansion to avoid co-product allocation, attributing all inputs and outputs to SM while accounting for the avoided production of marginal vegetable oils, primarily palm oil and, alternatively, rapeseed oil. When palm oil was considered the marginal product, SM exhibited a global warming potential of 721 g CO_2_ eq., 3.1 g SO_2_ eq. for acidification potential, −2 g NO_3_ eq. for eutrophication potential, 0.3 mg CFC11 eq. for ozone depletion potential, and 0.4 g ethene eq. for photochemical smog potential per kilogram, with a land occupation of 3.6 m^2^year per kg. Attributional analyses using economic and mass allocation identified global warming, eutrophication, and acidification as the dominant impact categories. Soybean cultivation emerged as the primary hotspot for climate impacts, driven by N_2_O emissions from crop residue degradation and biological nitrogen fixation, while soybean transport by truck contributed notably to eutrophication and acidification, with acidification potential showing high sensitivity to transport distance. While consequential LCA is a practical approach for SM, the analysis emphasized the need to consider associated production systems affected by soybean oil co-products, including palm oil, rapeseed, and spring barley [141].

The environmental implications of substituting SM with alternative protein sources have also been explored. In French dairy production systems, an LCA comparing feeding rations based on Brazilian-produced SM and locally produced rapeseed meal (RSM) across nine impact categories, including global warming, ecotoxicity, and eutrophication. The results showed that crop production was the dominant contributor to most environmental impacts, whereas overseas transport of SM had only a marginal influence. Under the specific conditions examined, continued reliance on imported SM resulted in lower overall environmental impacts than the use of domestically produced RSM. Notably, transport of SM from Brazil to France was not a significant contributor to climate change impacts, which reflects soybean cultivation systems characterized by low tillage intensity. Despite extensive pesticide and nitrogen fertilizer use, no-till practices reduced machinery demand and enhanced soil carbon stocks, partially offsetting other environmental burdens [142].

Nevertheless, Zanten et al. found that substituting SM with RSM in finishing pig diets led to marginal reductions in global warming potential and energy use, and up to 12% lower land use per kilogram of body weight. The extent of these benefits depended strongly on animal performance, with the lowest impact observed under scenarios of reduced growth and a higher body protein-to-lipid ratio that improved feed conversion efficiency. Direct and indirect land-use change emissions had minimal influence, and when only feed production was considered, replacing SM with RSM reduced global warming potential by up to 10%, energy use by up to 5%, and land use by up to 16% [143].

In addition to GHG emissions and land use, water consumption represents an additional environmental dimension in SM assessment. Although SM provides high protein density in poultry and swine diets, its cultivation is relatively water-intensive. Reducing SM inclusion can lower water use; however, substitutions with alternatives such as distillers dried grains with solubles (DDGS) and synthetic amino acids may increase GHG and energy demand. This trade-off illustrates the multifaceted nature of SM sustainability, where nutritional efficiency, water use, energy demand, and emissions must be evaluated simultaneously from an LCA perspective [144].

LCA results should be interpreted with consideration of methodological limitations and context-dependent trade-offs. Differences in system boundaries, functional units, allocation procedures, land-use change assumptions, and substitution scenarios can substantially influence estimated environmental impacts and the comparability of LCA results [145,146,147]. Moreover, improvements in one impact category may be accompanied by increases in others, highlighting the need to consider multiple environmental and economic dimensions simultaneously [140,141,142,143,144]. Therefore, comparisons among LCA studies should be made cautiously, particularly when production systems, geographical contexts, and formulation assumptions differ.

Overall, LCA studies indicate that the environmental performance of SM is highly sensitive to compositional quality, formulation strategy, system boundaries, and substitution context. Rather than yielding uniform conclusions, these assessments highlight the necessity of integrated, system-specific evaluations to balance nutritional benefits, economic feasibility, and environmental impacts when positioning SM within sustainable agri-food and livestock production systems.

## 8. Limitations, Safety, and Translational Barriers

Despite the considerable potential of SM and SM-derived peptides, several limitations remain to be addressed before their broader translation into food, nutraceutical, and industrial applications. Residual antinutritional factors may remain following processing and adversely affect nutrient digestibility and utilization, while soybean proteins are also important food allergens that require appropriate safety assessment and risk management [148,149]. The extent to which these factors are reduced depends on the processing method and conditions; moreover, intensive thermal processing may induce protein denaturation, Maillard reactions, and alterations in protein functionality [149,150]. Processing-related sensory limitations, particularly off-flavor formation, may further restrict the incorporation of soy-derived ingredients into food products, while conventional approaches for removing off-flavor precursors may compromise protein functionality [151].

For SM-derived bioactive peptides, gastrointestinal stability, absorption, and systemic bioavailability remain important translational challenges because bioactivities demonstrated in vitro do not necessarily translate into physiological effects in vivo. Although numerous soybean-derived peptides with antioxidant, antihypertensive, anti-inflammatory, and other biological activities have been identified, further studies are needed to establish their target tissues, mechanisms of action, and physiological relevance. Much of the evidence for the health effects of soybean-derived peptides remains based on in vitro and animal studies, whereas clinical evidence for specific peptides and well-defined mechanisms remains comparatively limited [152].

Variability in SM composition associated with soybean variety, geographical origin, environmental and cultivation conditions, storage, and processing can further complicate reproducibility, quality control, and standardization [153]. Finally, scaling optimized laboratory-scale processes to industrial production remains challenging because of process control, heat and mass transfer, raw-material variability, quality consistency, economic feasibility, and the lack of standardized large-scale processing systems, particularly for solid-state fermentation of SM [154]. Addressing these barriers through standardized processing, rigorous safety evaluation, improved understanding of peptide bioavailability, well-designed clinical studies, and scalable processing technologies will be essential for the successful advancement of SM-derived products toward practical applications.

## 9. Conclusions and Future Perspectives

SM represents a multifunctional protein resource that can be valorized through diverse applications ranging from conventional feed uses to human food, biotechnology, industrial, agricultural, and environmental sectors. Its abundant proteins, balanced amino acid composition, functional carbohydrates, minerals, and bioactive compounds provide a foundation for developing value-added products across diverse applications. In particular, SM proteins and derived hydrolysates serve as promising sources of bioactive peptides with antioxidant, antihypertensive, hepatoprotective, and other functional properties, while their carbohydrate and fiber fractions contribute prebiotic and water-retention functionalities.

Recent advances in processing technologies, microbial fermentation, and protein modification strategies have expanded the potential of SM as a sustainable raw material for functional foods, nutraceutical ingredients, microbial fermentation substrates, and bio-based industrial materials. Furthermore, agricultural and environmental applications, together with LCA approaches, highlight the role of SM valorization in promoting circular bioeconomy and resource-efficient production systems.

The selection of SM type and processing strategy should be application-specific. HM, which accounts for the majority of commercial production, is widely used in animal feed owing to its lower processing cost. However, the associated high-temperature treatment causes greater protein denaturation and reduces protein solubility. In contrast, LM undergoes less protein denaturation and retains higher protein solubility and dispersibility, making it more suitable for applications requiring greater protein functionality. For feed applications, fermentation is particularly promising for reducing ANFs and improving protein utilization, whereas enzymatic hydrolysis is more appropriate for the targeted production of bioactive peptides or functional ingredients. For industrial applications, physicochemical modification or crosslinking can be employed to tailor the structural and material properties of SM-based products. Overall, matching the SM type and processing strategy with the intended application and desired nutritional or functional properties is essential for effective SM valorization.

Future research should focus on optimizing targeted processing strategies to improve protein functionality, enhance bioactive peptide generation, and reduce antinutritional factors. Emerging peptide discovery approaches integrating LC-MS/MS-based peptidomics, molecular docking, molecular dynamics simulations, and artificial intelligence-assisted prediction are expected to facilitate the identification and rational design of multifunctional SM-derived peptides by revealing sequence–activity relationships, peptide–target interactions, and potential mechanisms of action. Integration of these advanced computational approaches with sustainable bioprocessing and environmental assessment will facilitate the transition of SM from a conventional agricultural by-product into high-value functional ingredients and industrially relevant bioproducts.

## Data Availability

No new data were created or analyzed in this study. Data sharing is not applicable to this article.
